# Development of a Dairy-Free Fermented Oat-Based Beverage With Enhanced Probiotic and Bioactive Properties

**DOI:** 10.3389/fmicb.2020.609734

**Published:** 2020-12-03

**Authors:** Liwei Chen, Daoyan Wu, Joergen Schlundt, Patricia L. Conway

**Affiliations:** ^1^School of Chemical and Biomedical Engineering, Nanyang Technological University, Singapore, Singapore; ^2^Nanyang Technological University Food Technology Centre (NAFTEC), Nanyang Technological University, Singapore, Singapore; ^3^Key Laboratory of Biological Resource and Ecological Environment of Chinese Education Ministry, College of Life Sciences, Sichuan University, Chengdu, China; ^4^Centre for Marine Science and Innovation, School of Biological, Earth and Environmental Sciences, The University of New South Wales, Sydney, NSW, Australia

**Keywords:** Lactobacillus fermentum PC1, oats, viability, in vitro digestion, antioxidant activity, phenolic content

## Abstract

*Lactobacillus fermentum* PC1 with proven probiotic properties was used to ferment oats with added honey to develop a probiotic beverage with enhanced bioactive ingredients. The viable *Lactobacilli* were enumerated during the fermentation and storage at 4°C, as well as after exposure to simulated gastrointestinal tract conditions. Good survival was noted both during storage as well as when exposed to the *in vitro* digestive tract conditions. Comparative analysis of the antioxidant activity, total phenolic content, and phenolic composition indicated fermentation improved the total antioxidant capacity and phenolic acid concentration. An increase of more than 50% of gallic acid, catechin, vanillic acid, caffeic acid, p-coumaric acid, and ferulic acid was observed in the methanol extracts. Moreover, no significant decrease in the β-glucan content was noted during fermentation and storage. In conclusion, this fermented product has a great potential as a functional food with enhanced probiotic survival and increased bioactive ingredients.

## Introduction

Probiotics can impart a range of beneficial effects including improving digestion, strengthening the immune system, and modifying the gut microbiome. It is recognized that the probiotic needs to be viable for maximum benefit, and it is generally accepted that the finished product should contain at least 10^6^–10^7^ viable cells per ml (CFU per ml; Shori, 2015). Consequently, there has been an increasing interest in improving the survival of probiotics in the finished product, during storage and when consumed and exposed to digestive tract conditions. Traditional fermented dairy foods are frequently used for probiotic delivery, because probiotic strains have been shown to survive well under these conditions. However, there is an increasing demand for non-dairy probiotic foods because of the rise in lactose intolerance, milk allergy, and an interest in low cholesterol content products (Gupta and Abu-Ghannam, 2012). Probiotic fermented non-dairy products have the advantage of being lactose free and having a low cholesterol content (Ranadheera et al., 2017). Cereals, fruits, and vegetable-based probiotic fermented products have received increasing attention in recent years because they can be alternatives to dairy based products and also because such products often contain complex carbohydrates that can be preferentially utilized by the probiotics (Verspreet et al., 2016). Such carbohydrates, referred to as prebiotics, can promote the growth of probiotics and, thereby, enhance the performance of the probiotic, for example, by inhibiting the growth of potentially pathogenic gut microbes (Verspreet et al., 2016).

In recent years, oats consumption has been linked to numerous health benefits, such as anti-inflammatory and antioxidant activity, and shown to have the potential to reduce the risk of cardiovascular diseases (CVD), type 2 diabetes, gastrointestinal disorders, and cancer (Martínez-Villaluenga and Peñas, 2017). Oats contains bioactive compounds, especially natural antioxidant phenolic compounds (Soycan et al., 2019) and β-glucan (Ho et al., 2016). Thus, oats is becoming a popular matrix of choice for innovative functional probiotic containing foods (Angelov et al., 2018). It has been shown that oats can promote the growth of lactic acid bacteria (Herrera-Ponce et al., 2014; Wu et al., 2018). In addition, there are reports of the optimization of the total phenolic content and antioxidant capacity in oats by fermentation using yeast or bacteria (Duru et al., 2019; Bei et al., 2020). Unfortunately, most studies did not achieve improvements in both probiotic and bioactive properties (Duru et al., 2019) or only focused on one aspect (Hole et al., 2012; Călinoiu et al., 2019; Bei et al., 2020).

Fermentation is the breakdown of carbohydrates, such as starch and sugar, by bacteria and yeast. It is an ancient technique for preserving food. Common fermented foods include kimchi, sauerkraut, kefir, tempeh, kombucha, and yogurt. More recently, many health benefits have been proposed for fermented foods and these included reducing heart disease risk, aiding digestion, and enhancing immunity and weight loss (Şanlier et al., 2019). The aim of this work was to develop a non-dairy fermented beverage that delivered novel fermentation products with both improved probiotic and bioactive properties. In preliminary studies, a significant decrease of the β-glucan content was found in oats when no sugar was added in the fermentation, most probably because the β-glucan is a selective substrate of *Lactobacilli* (Jaskari et al., 1998). Thus, we hypothesized that if we wanted to develop an oat-based probiotic food with both improved probiotic viability and bioactive ingredients, we needed to add sugars to promote the growth of the probiotic strain and enhance fermentation. With the target to develop a healthy functional food, honey was used as a sugar source for the probiotic strain, because honey can have prebiotic activity (Conway et al., 2010) and also contains antioxidant and oligosaccharides and, therefore, is a suitable ingredient of functional foods (Das et al. 2015). Moreover, it has been used in old and modern medical practice due to its antimicrobial, anti-inflammatory, and wound-healing properties (Al-Waili et al., 2011).

In our previous work, *Lactobacillus fermentum* PC1 has been shown to have good capacity for attenuating inflammation, working as an oral adjuvant and influencing the gut microbes (Plant and Conway, 2002; Plant et al., 2003; Esvaran and Conway, 2016; Esvaran and Conway, 2019). It has been shown to tolerate well bile salts and low pH and survive passage through the digestive tract when dosed at the high dose, but less well at a low dose (Gibson and Conway, 1994). Furthermore, we have previously shown that the addition of a prebiotic enhanced the survival of probiotics (O’Riordan et al., 2001). Thus, in this study, we aim to evaluate if the viability of *L. fermentum* PC1 during *in vitro* digestion and storage could be improved through the use of oats and honey as a delivery matrix fermented with *L. fermentum* PC1, and if such fermentation could enhance and maintain the bioactive ingredients in the end product. In addition to the viability testing, the sugar consumption and organic acid production were monitored during fermentation and storage. We also evaluated the bioactive ingredients including antioxidant potential, total phenolic acid content, phenolic composition, and β-glucan.

## Materials and Methods

### Materials, Enzymes, and Strain

High performance liquid chromatography (HPLC)-grade formic acid, acetonitrile, and Folin-Ciocalteau reagent as well as phenolic acid standards including gallic acid, chlorogenic acid, Catechin, 4-hydroxybenzoic acid, caffeic acid, vanillic acid, p-coumaric acid, sinapic acid, ferulic acid, and quercetin were all purchased from Sigma-Aldrich (Singapore).

Whole grain oat flour from Bob’s Red Mill was purchased from Lazada online shop in Singapore. *L. fermentum* PC1 (FII511400) was obtained from the CRC Food Industry Innovation culture collection. Human α-amylase (A1031), porcine pepsin (P6887), porcine trypsin (T4799), bovine chymotrypsin (C4129), porcine pancreatic lipase (L0382), and fresh bile salts (B8756) were purchased from Sigma-Aldrich (Singapore). DeMann-Rogosa-Sharpe (MRS) broth and agar were obtained from Sigma-Aldrich (Singapore) and prepared according to the manufacturer’s instructions.

### Fermentation Conditions

Dry oat flour was autoclaved at 121°C for 10 min. Honey from Sardinia (Miele Di Sardegna honey) was suspended in distilled water (3 g honey in 90 ml distilled water) and pasteurized at 80°C for 10 min. The diluted honey was added aseptically to the sterilized oat flour to yield a final concentration of 10% oats (w/v) and 3% honey (w/v). This mixture was heated to 80°C for 10 min with regular stirring in a thermostatically controlled water bath to ensure homogenization. The mixture was cooled to room temperature before inoculation.

Overnight-grown *L. fermentum* PC1 strain in MRS (pH 6.2 ± 0.2) was inoculated into the oat and honey mixture at 1% (v/v) to yield an initial concentration of about 10^7^ per ml. The mixture was fermented in screw cap bottles (250 ml) at 37°C, 150 rpm for 72 h, and subsequently stored at 4°C for 14 days. Samples were taken daily during fermentation and then, after 10 and 14 days of storage, analyzed for viable count of *Lactobacilli*, pH values, and bioactive compounds as well as viable counts after exposure to simulated digestive tract conditions.

### Enumeration of Viable *L. fermentum* PC1

Viable *Lactobacilli* in the fermented product were quantified using the standard plate count method (Conway et al., 1987). In brief, 1 ml of fermented product was used to make 10-fold serial dilutions in PBS. Aliquots of 10 μl of appropriate dilutions were plated in triplicate on MRS agar plates using the drop plate method. The plates were incubated at 37°C for 48 h. Colonies were counted and recorded as log CFU (colony forming units) per ml.

### Impact of Simulated Digestive Tract Conditions on Survival of *L. fermentum* PC1 in Fermented Oat

Fermented oat products were exposed to conditions which simulated oral, gastric, and small intestinal digestion conditions according to a published method with slight modifications (Minekus et al., 2014). *L. fermentum* PC1 48 h secondary culture grown in MRS was washed and re-suspended in PBS to about 10^7^ per ml and used as control. In summary, samples were initially combined with simulated salivary fluid with a final concentration of human α-amylase of 75 U per ml, and the mixture was incubated for 2 min at pH 7, followed by the addition of simulated gastric fluid with final concentration of porcine pepsin of 2000 U per ml and pH 3, and incubated for 2 h. The mixture was then combined with simulated intestinal fluid and incubated for another 2 h after pH adjustment to 7 and with final concentrations of the following enzymes: porcine trypsin (100 U per ml), bovine chymotrypsin (25 U per ml), porcine pancreatic lipase (2000 U per ml), and fresh bile salts (10 mM). All three steps were performed at 37°C. Aliquots (100 μl) of undigested samples, and samples taken after simulated gastric phase and intestinal phase were serially diluted. Aliquots (10 μl) of appropriate dilutions were plated on MRS agar plates using the drop plate method for enumeration of viable *Lactobacilli*.

### Glucose, Fructose, Lactic Acid, and Acetic Acid Assay

Samples taken throughout the experiment were centrifuged at 16,000 × *g* for 30 min, and then supernatants diluted three or four times in 5 mM H_2_SO_4_ prior to filtration using a 0.45 μm membrane (PES, VWR). Glucose, fructose, lactic acid, and acetic acid concentrations in the supernatant were determined using an Ultra-Fast Liquid Chromatography (Shimadzu) equipped with a refractor index detector (RID-10A). An Aminex® HPX-87H column (Bio-Rad, Singapore) was used for the separation with 5 mM H_2_SO_4_ as the mobile phase at a flow rate of 0.6 ml/min according to the manufacturer’s instructions. The temperature of the column oven and RID were set at 50°C and 45°C, respectively. Samples (20 μl) were injected in duplicate for each independent experiment. Concentration of glucose, fructose, lactic acid, and acetic acid were calculated according to a standard curve prepared using concentrations ranging from 0.625 to 20 g/L.

### Ultrasound-Assisted Extraction of Phenolic Compounds

Samples were extracted using a slightly modified variant of the published ultrasound-assisted extraction method (Călinoiu et al., 2019). Firstly, 40.0 ± 0.1 mg of freeze-dried sample was accurately weighed, and 1.8 ml of hexane was added to remove fats. The mixtures were vortexed for 30 s, sonicated for 10 min, and vortexed for another 30 min. Then, the mixture was centrifuged for 15 min at 8,000 × *g*, the supernatant was discarded, and the wet samples were dried for 30 min at 30°C in a fume hood. These dried samples were extracted by adding 80:20 methanol:water (1.5 ml) and vortexed until fully suspended and held in a sonic bath for 1 h at 40°C. Samples were then vortexed for another 20 min prior to being centrifuged for 15 min at 10,000 × *g*. This extraction was repeated one more time and the supernatants were combined and evaporated to dryness. The dried extracts were reconstituted in 0.2 ml 80% methanol, vortexed for 5 min, and then centrifuged in 10,000 × *g* for 20 min prior to analyses of antioxidant activity, total phenolic content, and HPLC analysis of phenolic compounds.

### Determination of Antioxidant Activity and Total Phenolic Content

Total antioxidant activity was analyzed by Total Antioxidant Capacity Assay Kit (Sigma-Aldrich, MAK187) according to the manufacturer’s instructions. Briefly, 5 μl of the methanol extracts were mixed with Cu^2+^ reagent and incubated in darkness at room temperature for 90 min, and the absorbance was measured at 570 nm using a microplate reader (Bio-Rad, Benchmark Plus Microplate Spectrophotometer System). Trolox solutions ranging from 0 to 20 nmol per well were used to prepare a standard curve. The antioxidant activity was expressed as nmol Trolox equivalents per mg sample (nmol TE/mg).

Total phenolic content was analyzed according to the Folin-Ciocalteu method with modification (Călinoiu et al., 2019). Briefly, 20 μl phenolic extract was mixed with 10 μl Folin-Ciocalteu’s reagent for 5 min. Then, 30 μl 20% Na_2_CO_3_ (w/v) and 140 μl of distilled water were added to the solution to reach a final volume of 200 μl. The mixture was incubated in the dark for 60 min at 300 rpm at room temperature. The plate was centrifuged at 200 × *g* and 120 μl samples of supernatant from each well were transferred to a new plate, and the absorbance was read at 760 nm with a microplate reader (Bio-Rad, Benchmark Plus Microplate Spectrophotometer System). A standard curve was prepared using a series of concentrations of gallic acid ranging from 0 to 8.4 μg per well. The results were expressed as mg gallic acid equivalents per g sample (mg GAE/g).

### HPLC Analysis of Phenolic Compounds

The HPLC analyses were carried out using an Agilent 1290 Infinity LC system coupled with photodiode array detector. Separation was performed at 25°C on a ZORBAX RRHD SB-C18 column (1.8 μm, 2.1 mm × 150 mm; Agilent Technologies, Singapore). Two solvents were used for the mobile phase: 0.1% formic acid in distilled water (v/v; solvent A) and 0.1% formic acid in acetonitrile (v/v; solvent B). The following optimized gradient elution (expressed in % B) was used: 0–2 min, 5% B; 2–6 min, 5–14% B; 6–38 min, 14–40% B; 38–40 min; 40–90% B; 40–42 min, 90–5% B; 40–45 min, 5% B. Aliquots (20 μl) of phenolic extracts from each time point were injected into the column. The flow rate was 0.3 ml/min, and detection was performed at 280 nm. Phenolic acids were identified by comparing their retention times and UV visibility with the standards under same analysis conditions. Quantitation was based on linear calibration curves of phenolic acid standards prepared using concentrations ranging from 0.78125 to 100 mg/L. All measurements were performed in triplicate and all the samples were injected in duplicate. The final concentrations of phenolic acids were expressed as μg/g.

### Analysis of β-Glucan

The β-glucan content in the fermented oat product was quantified using the Mixed Link (1–3, 1–4) Beta Glucan kit (Megazyme International, Bray, Ireland) with modifications of method B. In brief, 30 mg (±1%) of freeze-dried fermented oat product was weighed to 0.1 mg precision and transferred into a 2 ml plastic screw cap tube. Firstly, the sample was extracted with 1.75 ml of 50% (v/v) aqueous ethanol to remove free sugars and fats. The extraction was repeated two additional times and the supernatant after centrifugation was discarded. Secondly, the pellet was suspended in 1.0 ml of sodium phosphate buffer (20 mM, pH 6.5) and the tube was incubated at 50°C for 5 min. Thirdly, 50 μl of lichenase (2.5 U) was added and the tube was vortexed and incubated for 1 h at 50°C with stirring at 300 rpm. Then, 0.5 ml of sodium acetate buffer (200 mM, pH 4.0) was added and the mixture was vigorously mixed. After that, the tubes were centrifuged for 10 min at 10,000 × *g*. Aliquots (25 μl) were transferred into 2 ml test tubes, and β-glucosidase (25 μl, 0.05 U) in 50 mM sodium acetate buffer (pH 4.0) was added and then the tubes were incubated at 50°C for 10 min. Finally, GOPOD Reagent (0.75 ml) was added to each tube prior to incubation at 50°C for a further 20 min. Glucose concentrations in the samples were measured at 510 nm against a reagent blank using SPECTRONIC 200 (Thermo Scientific, Singapore). Reagent blanks and D-glucose standards of 1 mg/ml were included in duplicate. For every independent assay, the test was carried out in duplicate with a reaction blank. The final β-glucan content was expressed as g/100 g dry weight (DW).

### Statistical Analysis

All the experiments were performed at least as three independent experiments, each analyzed in duplicate. The results are expressed as mean ± SD. Statistical analyses were carried out using either the Student’s *t*-test or one-way ANOVA in R (version 3.6.3). Values of *p* < 0.05 were considered statistically significant. Correlations between total phenolic content and antioxidant activity were determined using Pearson’s correlation. Correlation coefficient *r* > 0.5 is considered to show a strong positive correlation.

## Results and Discussion

### *L. fermentum* PC1 Growth During Fermentation

In this study, whole grain oat flour was used as a delivery vehicle for *L. fermentum* PC1 by fermenting 10% oat flour supplemented with 3% honey in distilled water with no additional ingredients. *L. fermentum* PC1 was inoculated around 10^7^ cell per ml. The growth profile shown in Table 1 demonstrated that there was a significant increase of viable PC1 cells during the first 24 h (7.96 log CFU/ml) as compared to 0 h (7.12 log CFU/ml), with a slight decrease after 48 h and 72 h to 7.28 log CFU/ml and 7.38 log CFU/ml, respectively. The viable count remained relatively stable during storage at 4°C for 10 days (7.40 log CFU/ml) and 14 days (7.32 log CFU/ml). With this increase of cell growth, there was a significant decrease of pH from 6.26 to 4.12 after 24 h, and a further decrease to 3.93 at 72 h. The pH of the fermented product was constant during storage at 4°C, which reflects the noted stability of organoleptic properties of the product. The stability was probably due to the buffering capacity of other compounds produced in the fermented product, such as acetic acid, lactic acid, and phenolic compounds. These results demonstrated that oat flour and honey supported the growth of *L. fermentum* PC1 and maintained viability during storage at 4°C, with levels above the recommended concentration of 10^6^–10^7^ CFU per ml (Shori, 2015).

**Table 1:** Viability of *Lactobacillus fermentum* PC1 and pH value in the fermentation product.

Parameter	Fermentation and storage time	Viable count
Viable count (log CFU/ml)	0 h (day 0)	7.12 ± 0.04^a^
	24 h (day 1)	7.96 ± 0.05^b^
	48 h (day 2)	7.28 ± 0.03^c^
	72 h (day 3)	7.38 ± 0.01^d^
	storage 10-day at 4°C (day 13)	7.40 ± 0.03^d^
	storage 14-day at 4°C (day 17)	7.32 ± 0.06^c^
pH	0 h (day 0)	6.26 ± 0.02^a^
	24 h (day 1)	4.12 ± 0.01^b^
	48 h (day 2)	4.05 ± 0.01^c^
	72 h (day 3)	3.93 ± 0.02^d^
	storage 10-day at 4°C (day 13)	3.94 ± 0.01^d^
	storage 14-day at 4°C (day 17)	3.96 ± 0.01^d^

Results are presented as mean ± SD. Values in the column with different superscript letters (a–d) are significantly different (*p* < 0.05).

Similarly, probiotic *L. casei* fermented with different oat substrates, including simple and germinated oat, had a final cell growth from 6.3 to 7.12 log CFU/ml (Herrera-Ponce et al., 2014). It has been shown that different fermentation factors including the percent of oats, sugar content, inoculum size, and fermentation time all influence the growth and stability of lactic acid bacteria in these products (Gupta et al., 2010). These workers optimized conditions and showed that with 5.5% oats, 1.25% sugar, and 5% inoculum (9.34 log CFU/ml) and a short fermentation time of 8 h, a high growth of 10.4 log CFU/ml *L. plantarum* ATCC 8014, was obtained; however, there was a reduction of viability of about 0.5 log CFU/ml at 14 days and 0.9 log CFU/ml at 21 days during storage at 4°C (Gupta et al., 2010). For the development of functional foods, not only high cell viability reached during fermentation is critical for maintaining the function of the probiotic and the desired level of acid production, but the stability of viable cells during storage is also important for maintaining the quality of the products. Our results demonstrated that this oat flour and honey fermented product has a great potential for the delivery of viable *L. fermentum* PC1 cells.

### Survival of *L. fermentum* PC1 in Simulated Digestive Tract Conditions

**Figure 1:**
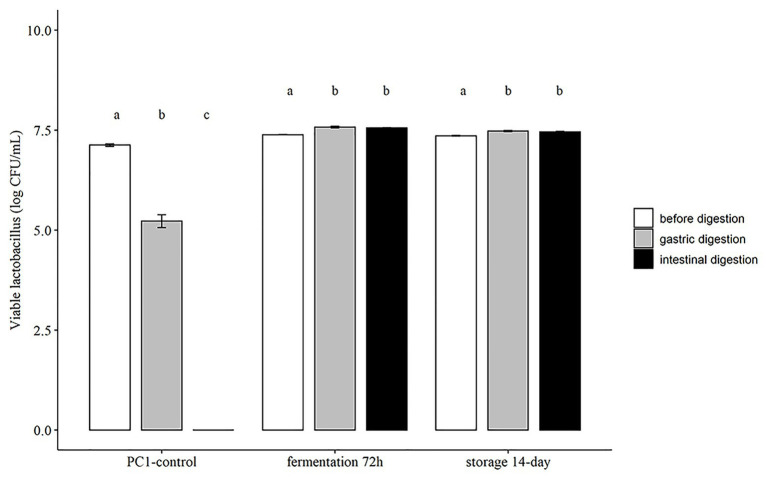
Survival of *Lactobacillus fermentum* PC1 during exposure to simulated gastrointestinal conditions. Results are expressed as Log CFU/ml in mean ± SD. Values of *p* were calculated using *t*-test. The statistics are presented by labeling lowercase letter “a, b, c.” The different superscripted letters indicate significant (*p* < 0.05) differences between each other.

The tolerance to gastrointestinal conditions is important for the function of probiotic strains in the gut. There are many different food matrices that have been investigated as probiotic carriers, but only a few studies have evaluated the effect of gastrointestinal conditions on the survival capacity of probiotics (Swieca et al., 2018). With the aim to improve the tolerance of *L. fermentum* PC1 under gastrointestinal conditions, we investigated the survival rate of *L. fermentum* PC1 in the fermented oat product using a standardized *in vitro* method for simulating conditions in the digestive tract (Minekus et al., 2014). The results indicate that both fermented products harvested at 72 h, and the product stored at 4°C for 14 days had significantly higher viable count and recovery rate as compared to control *L. fermentum* PC1 culture grown in MRS and resuspended in PBS (Figure 1). For the control *L. fermentum* PC1 48 h culture suspended in PBS, a viable count of 5.22 ± 0.28 log CFU/ml was obtained after simulated saliva and gastric conditions, while the subsequent intestinal simulated conditions resulted in no detectable viable cells (<3 log CFU/ml). In contrast, for *L. fermentum* PC1 fermented oat product, viable counts of 7.57 ± 0.05 log CFU/ml and 7.55 ± 0.03 log CFU/ml after exposure to simulated gastric and intestinal conditions were observed, respectively. For fermented oat product stored at 4°C for 14 days, viable counts of 7.48 ± 0.04 log CFU/ml and 7.46 ± 0.03 log CFU/ml after exposure to simulated gastric and intestinal conditions were observed, respectively (Figure 1).

Several studies have reported the improved survival rate of probiotic strains in food carriers. The protection effect depends on the probiotic strains, food matrices used, and fermentation conditions. For example, the inclusion of milk has been shown to significantly improve survival of probiotic *Lactobacilli* when exposed to simulated digestive tract conditions (Lo Curto et al., 2011). This is consistent with an earlier clinical study showing that drinking milk enhanced the survival of probiotic strains and raised the pH in the stomach (Conway et al., 1987). Another study used legumes with *L. plantarum* 299v and obtained a recovery rate of above 80% for lentils and around 40% for mung beans (Swieca et al., 2018). The difference in recovery rate is specific for the strains and the food matrices used. The high buffering capacity of oat flour and honey could probably be one important factor contributing to the high survival rate of the PC1, as previously shown for milk (Conway et al., 1987; Lo Curto et al., 2011). Moreover, sugar residues after fermentation could be another important factor that contributes to the survival rate noted in the present study, since it has been shown that survival of *Lactobacilli* in acidic environments is enhanced in the presence of metabolizable sugars (Corcoran et al., 2005).

### Sugars and Organic Acids Content During Fermentation and Storage

**Figure 2:**
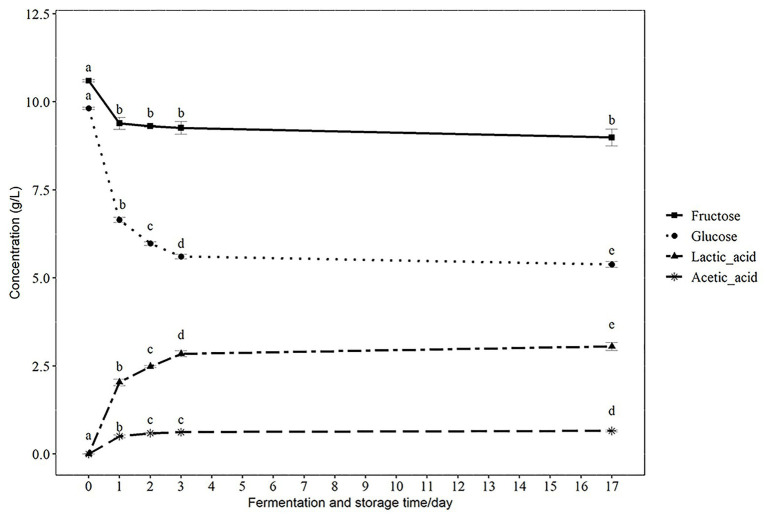
The concentration of glucose, fructose, lactic acid, and acetic acid during fermentation and storage. Results are presented as g/L in mean ± SD. Values of *p* were calculated using *t*-test. The statistics are presented by labeling lowercase letter “a, b, c, d, e.” The different superscripted letters indicate significant (*p* < 0.05) differences between each other.

The compositions of sugars and organic acids are important indicators of the metabolic state of probiotics during fermentation and storage. Since lactic acid is the major end-product of carbohydrate metabolism by lactic acid bacteria (Abdel-Rahman et al., 2013), the observed decrease in glucose and fructose and increase of lactic acid during fermentation were expected. In addition, we observed an increase in the concentration of acetic acid during fermentation (Figure 2). There was a significant decrease of glucose from 9.82 ± 0.03 g/L at 0 h to 6.65 ± 0.07 g/L at 24 h and a further decrease to 5.61 ± 0.07 g/L at the end of fermentation (72 h) with 5.38 ± 0.09 g/L after14 days of storage (Figure 2) at 4°C. The concentration of fructose decreased from 10.6 ± 0.03 g/L at 0 h to 9.39 ± 0.17 g/L after 24 h, with no significant decrease during further fermentation and storage. The concentration of lactic acid significantly increased from 0 g/L at 0 h to 2.04 ± 0.09 g/L at 24 h, and further increased to 2.85 ± 0.08 g/L after 72 h fermentation. There was a slight increase of lactic acid to 3.06 ± 0.11 g/L after 14 days of storage at 4°C (Figure 2). The concentration of acetic acid increased from 0 g/L at 0 h to 0.51 ± 0.04 g/L after 24 h and increased further to 0.62 ± 0.02 g/L by 3 days of fermentation and to 0.66 ± 0.03 g/L after storage at 4°C for 14 days (Figure 2). The observed sugar consumption and acid production were consistent with the growth of the *Lactobacillus* and decrease of pH value. The change of sugar and acids will contribute to the flavor and taste of the final product. Similar changes of sugar and organic acids were also observed in other lactic acid bacteria fermented products, such as fermented apple juice and coconut water beverage (Giri et al., 2018; Li et al., 2018). Similar to these studies, we also observed a substantial amount of residual glucose and fructose (>50%) in the fermented products, even after 3 days of fermentation. Moreover, a further decrease of glucose and an increase of lactic and acetic acids were observed in our product during storage at 4°C, which implies that there was ongoing metabolic activity of the *Lactobacillus* PC1 strain. Some studies indicated that the presence of residual sugars can assist in the continuous metabolic activity of the probiotics in fermented foods (Camargo Prado et al., 2015; Giri et al., 2018) and enhance the survival of *Lactobacilli* in acidic environments (Corcoran et al., 2005). This is consistent with the stable viability of the PC1 strain during storage and in simulated gastrointestinal conditions. This observation also suggests that metabolizable sugar could be one factor contributing to survival of the PC1 during storage and simulated gastrointestinal conditions.

### Antioxidant Activity and Total Phenolic Acid Content During Fermentation and Storage

**Figure 3:**
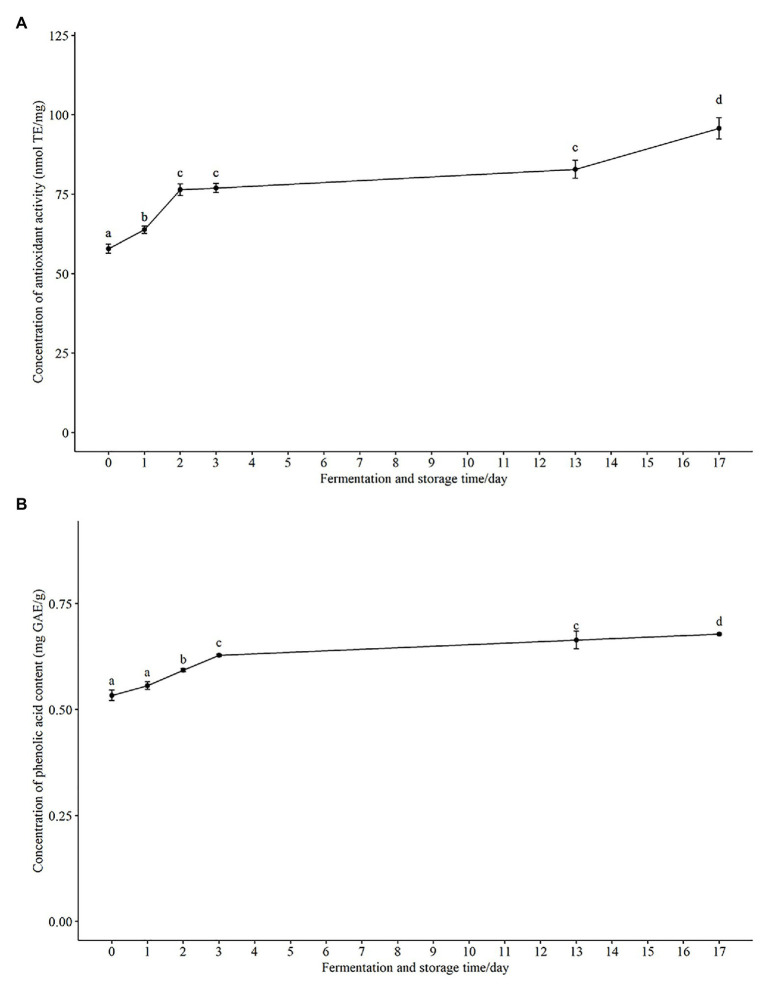
The change of total antioxidant activity and phenolic acid content in fermented product during fermentation and storage. **(A)** Total antioxidant activity are expressed as nmol Trolox equivalents per mg sample (nmol TE/mg). **(B)** Total phenolic acids are expressed as mg gallic acid equivalents per g sample (mg GAE/g). Results are presented as mean ± SD. Values of *p* were calculated using *t*-test. The statistics are presented by labeling lowercase letter “a, b, c, d.” The different superscripted letters indicate significant (*p* < 0.05) different between each other.

The health benefits of oats and honey have been associated with the presence of antioxidant capacity (Das et al., 2015; Martínez-Villaluenga and Peñas, 2017). Several studies have reported an enhanced antioxidant capacity and related bioactive compounds such as phenolic acids after fermentation (Hole et al., 2012; Călinoiu et al., 2019; Bei et al., 2020). In our study, 80% aqueous methanol (Călinoiu et al., 2019) was used to extract the methanol soluble antioxidant and phenolic components in the fermented oat product. An increased antioxidant activity was shown using the Cu^2+^ reagent-based antioxidant assay and results expressed as nmol Trolox equivalents per mg sample (nmol TE/mg; Figure 3A). The antioxidant activity increased significantly in the fermented product after both 24 h (63.8 ± 2.76 nmol TE/mg) and 48 h (76.4 ± 4.51 nmol TE/mg), compared with that measured at 0 h (57.9 ± 3.52 nmol TE/mg). There was no significant increase at 72 h (76.9 ± 3.49 nmol TE/mg) as compared with 48 h (76.4 ± 4.51 nmol TE/mg). Interestingly, there was a significant increase in antioxidant activity during storage at 4°C, and it reached 95.7 ± 8.07 nmol TE/mg after 14 days of storage (Figure 3A), which further supports the suggestion that the probiotic strain was metabolically active during the storage period.

In agreement with the increase of total antioxidant capacity, we observed an increase in the total phenolic content from 0.534 ± 0.03 mg GAE/g at 0 h to 0.628 ± 0.01 mg GAE/g after 72 h fermentation (Figure 3B). The total phenolic content continued to increase to 0.678 ± 0.01 mg GAE/g during storage for 14 days at 4°C (Figure 3B) as did the total antioxidant activity. Phenolic compounds have been reported to be one of the most important natural antioxidants in oats (Soycan et al., 2019) and honey (Conway et al., 2010), consequently the observed increase of both total phenolic content and total antioxidant capacity would be anticipated. Pearson’s correlation coefficient (r) between antioxidant activity and total phenolic content was 0.88. The strong positive correlation suggests that phenolic acid components present in the methanol extracts have a major contribution to the antioxidant activity of the fermented oat product. Similarly, solid-state fermentations of oats with fungi or other lactic acid bacteria have been shown to improve the phenolic composition and antioxidant activity of oats. For example, improved bioavailability of phenolic acids in barley and oats was observed during fermentation with eight probiotic strains (Hole et al., 2012). These workers showed that the improvement of phenolic acids was strain dependent and reported a dramatic increase of free phenolic acids (more than 25 folds) for three probiotic strains, *L. acidophilus*, *L. johnsonii*, and *L. reuteri* (Hole et al., 2012). An 83% increase of total phenolic content was observed in another solid-state yeast fermentation study (Călinoiu et al., 2019). Several probiotic fermentation studies have shown that the increase of antioxidant activity of fermented plant-based food is because there is increased release or synthesis of antioxidant compounds during fermentation (Hur et al., 2014; Călinoiu et al., 2019). The possible enzymes, such as glycoside hydrolase, cellulose, esterase, β-glucosidases, produced by strains during fermentation could enhance the availability of phenolics and other antioxidant compounds (Hole et al., 2012; Călinoiu et al., 2019). During fermentation, these enzymes could potentially release esterified and insoluble-bound phenols in a time-dependent manner (Călinoiu et al., 2019), because the enzyme production is dependent on fermentation time. Our data also indicated that the increased total phenolic content and antioxidant activity were dependent on fermentation time. In future studies, parameters including fermentation time could be optimized to enhance the total antioxidant capacity and total phenolic content. In addition, in order to identify the key enzymes in enhancing the availability of the phenolic content, it will be necessary to determine the changes of relevant enzyme. In short, our results support the finding that fermentation is an effective way to enhance the total antioxidant capacity of the probiotic product.

### Changes in Phenolic Composition During Fermentation

Significantly enhanced total and specific phenolic compounds in oats during solid state fermentation have been reported in several studies (Călinoiu et al., 2019, Bei et al., 2020); however, there are limited studies of improvement of specific phenolic compounds in liquid state fermentation. To assess the effect of *L. fermentum* PC1 fermentation on the bioavailability of specific phenolic acids in the fermented oat product, the concentrations of 10 phenolic acids in the methanol soluble extract during fermentation and storage were analyzed by HPLC. As shown in Table 2, the phenolic composition was influenced by fermentation. Of the 10 identified phenolic compounds, the levels of gallic acid, catechin, vanillic acid, caffeic acid, p-coumaric acid, ferulic acid, and sinapic acid increased during fermentation, with the highest relative increase occurring after 72 h fermentation (gallic acid +58.65%, catechin +92.35%, vanillic acid +67.17%, caffeic acid +117.08, p-coumaric acid +197.87, ferulic acid +116.35, sinapic acid +49.20%). There were no significant changes in the concentration of 4-hydroxybenzoic acid, chlorogenic acid, and quercetin during fermentation. There were significant decreases in the concentration of 4-hydroxybenzoic acid (−10.37%), vanillic acid (−55.02%), caffeic acid (−21.41%), p-coumaric acid (−52.81%), and ferulic acid (−9.02%) during storage for 14 days at 4°C but not below the initial value noted for caffeic acid, p-coumaric acid, and ferulic acid in the control at 0 h. Since the production and activity of possible enzymes involved in the liberation of phenolic compounds is dependent on fermentation time, the changes of phenolic acid production could be explained by the changes of enzyme production and stability (Călinoiu et al., 2019). Moreover, the modulation of phenolic content is highly depended on the microorganisms used. Since the enzymes responsible for the metabolism of phenolic compounds may be only expressed in specific strains (Adebo and Gabriela Medina-Meza, 2020), it has been shown that the improvement of phenolic acids (caffeic, p-coumaric, ferulic, and sinapic acids) vary between different probiotic strains (Hole et al., 2012), and changes are associated with the bacterial feruloyl esterase in different strains. The decrease of phenolic acids during storage could be related to the decline of available nutrient and accumulation of waste compounds in the product (Călinoiu et al., 2019). The observed phenolic compounds in our study are mostly in line with other reports, but the concentrations of some phenolic acids differ from other findings. For example, ferulic acid was reported as the major component in several studies (Soycan et al., 2019; Bei et al., 2020), but the concentration of ferulic acid detected here was lower than the level reported in other studies. Since the phenolic composition varies between different oat products and extraction methods (Călinoiu et al., 2019), this could be due to different oat flour and extraction method used. In addition, the added honey (Cheung et al., 2019) also contributed to the phenolic composition of our product. Overall, it was demonstrated that the fermented samples had higher concentrations of several individual phenolic acids as compared with the 0 h non-fermented control.

**Table 2:** Phenolic compounds analysis during fermentation and storage.

Phenolic compound	0 h	24 h	48 h	72 h	Storage 10-day	Storage 14-day
Gallic acid	2.93 ± 0.08^a^	3.28 ± 0.12^b^	3.62 ± 0.06^c^	4.64 ± 0.24^d^	4.81 ± 0.24^d^	4.76 ± 0.35^d^
4-Hydroxybenzoic acid	0.94 ± 0.08^a^	0.91 ± 0.06^a^	0.88 ± 0.08^a^	0.90 ± 0.03^a^	0.83 ± 0.05^b^	0.81 ± 0.05^b^
Chlorogenic acid	1.52 ± 0.23^a^	1.49 ± 0.07^a^	1.57 ± 0.10^a^	1.55 ± 0.11^a^	1.54 ± 0.02^a^	1.53 ± 0.07^a^
Catechin	1.27 ± 0.30^a^	1.93 ± 0.27^b^	2.22 ± 0.11^b^	2.43 ± 0.23^c^	2.37 ± 0.22^c^	2.43 ± 0.23^c^
Vanillic acid	0.34 ± 0.01^a^	0.38 ± 0.07^a^	0.47 ± 0.05^b^	0.56 ± 0.04^c^	0.48 ± 0.13^b^	0.25 ± 0.06^d^
Caffeic acid	0.39 ± 0.19^a^	0.65 ± 0.01^b^	0.73 ± 0.04^c^	0.85 ± 0.06^d^	0.68 ± 0.02^e^	0.67 ± 0.02^e^
p-Coumaric acid	0.45 ± 0.05^a^	0.47 ± 0.02^a^	1.24 ± 0.26^b^	1.33 ± 0.16^b^	0.82 ± 0.16^c^	0.63 ± 0.07^d^
Ferulic acid	0.32 ± 0.20^a^	0.60 ± 0.01^b^	0.58 ± 0.05^b^	0.70 ± 0.08^c^	0.61 ± 0.07^c^	0.64 ± 0.11^c^
Sinapic acid	1.42 ± 0.34^a^	1.56 ± 0.13^a^	1.82 ± 0.29^a^	2.12 ± 0.28^b^	2.15 ± 0.19^b^	2.14 ± 0.20^b^
Quercetin	5.08 ± 0.15^a^	5.25 ± 0.11^a^	5.37 ± 0.09^a^	5.47 ± 0.18^a^	5.43 ± 0.19^a^	5.45 ± 0.17^a^

Results are expressed as mean ± SD, Values in the same row followed by different superscript letters (a–e) indicate significant differences (*p* < 0.05) between days of fermentation and storage.

### Content of β-Glucan During Fermentation

**Figure 4:**
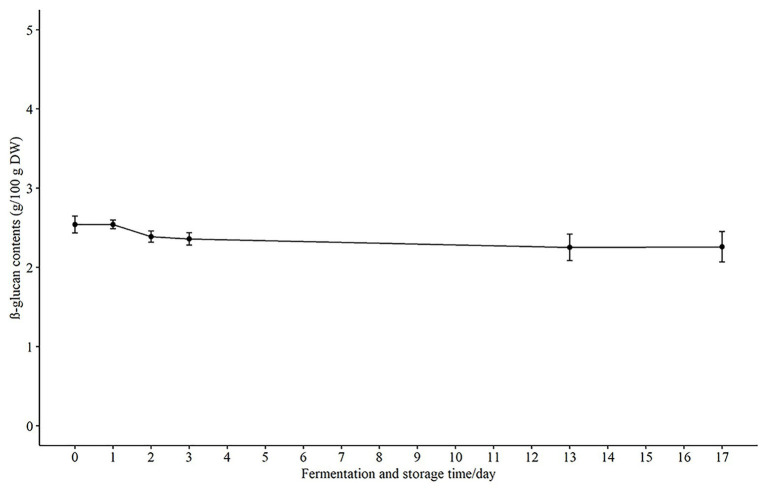
The concentration of β-glucan in fermented product during fermentation and storage. Results are presented as g/100 g DW (dry weight) in mean ± SD.

Another main bioactive component in oats, β-glucan, has cholesterol-lowering effects at dietary intake levels of at least 3 g per day and may reduce the risk of cardiovascular disease (Ho et al., 2016). It has been reported that there was a loss of β-glucan during harsh processing, such as excessive heat and shearing (Zhu et al., 2016). Therefore, it was of interest in the present study to follow the β-glucan levels during the fermentation and storage to ensure the β-glucan was not lost. There was no change in the β-glucan content during the first 24 h of fermentation with 2.54 ± 0.09 g/100 g DW as compared to 0 h (2.54 ± 0.185 g/100 g DW). There was a slight decrease (*p* > 0.05) to 2.36 ± 0.135 g/100 g DW after 3 days of fermentation, and this level was maintained at 2.26 ± 0.270 g/100 g DW during storage at 4°C for 14 days (Figure 4). These results are in agreement with another study that investigated the effect of fermentation on β-glucans in oat sourdough (Lu et al., 2019). These workers reported that the total β-glucan content decreased slightly during fermentation and was stabilized when the lactic acid bacteria counts were almost stable. β-glucan is selectively utilized by bifidobacteria and Lactobacilli in the gut (Jaskari et al., 1998), and thereby produce short chain fatty acids (SCFA) which are linked to beneficial physiological effects (Simpson and Campbell, 2015). Consequently, it is important to retain stable levels of the β-glucan to ensure the fermented oat product has the capacity to support growth of potentially beneficial bacteria and the production of SCFAs.

## Conclusion

In summary, it is shown that oat flour with added honey promoted the growth and maintained the survival of *L. fermentum* PC1 both during fermentation and storage. The viable count of PC1 was stable (7.32 log CFU/ml) during storage at 4°C for at least 14 days. The survival of the PC1 in the fermented oat exposed to simulated gastrointestinal conditions was significantly enhanced compared to control cells. Furthermore, it was apparent that the PC1 was metabolically active during storage at 4°C for at least 14 days since the content of sugars and acid production continued to change. Moreover, there were improvements in antioxidant capacity and phenolic content and no significant decrease of β-glucan. The main phenolic components that were detected in higher amounts in the methanol extracts were gallic acid, catechin, vanillic acid, caffeic acid, p-coumaric acid, ferulic acid, and sinapic acid. Thus, this study demonstrated that fermentation of oat flour with added honey and *L. fermentum* PC1 could be a potentially valuable probiotic food with both improved levels of probiotic and bioactive components.

## Data Availability Statement

The original contributions presented in the study are included in the article/supplementary material, further inquiries can be directed to the corresponding author.

## Author Contributions

LC and DW have contributed equally to this work in participating in the design of the study, analysis, and interpretation of the data and drafted the manuscript. JS participated in interpretation of the data and revised the draft critically. PC participated in conception and design of the study, analysis, and interpretation of the data and revised the draft critically. All authors accepted and approved the submitted version.

### Conflict of Interest

The authors declare that the research was conducted in the absence of any commercial or financial relationships that could be construed as a potential conflict of interest.
